# A New Species of the *Agriotes nuceus* Species Group from Turkey

**DOI:** 10.1673/031.013.1701

**Published:** 2013-03-15

**Authors:** Mahmut Kabalak, Osman Sert, İnanç Özgen, Giuseppe Platia

**Affiliations:** 1 Hacettepe University Faculty of Science, Department of Biology, Section of Applied Biology, 06532 Beytepe, Ankara, Turkey; 2 Firat University, Vocational School of Baskil, Department of Orchard Agriculture, Elaziğ, Turkey; 3 Via Molino Vecchio, 21/a, 47043 Gatteo (FC), Italy

**Keywords:** Elateridae, Elaterinae

## Abstract

A new Elateridae species, ***Agriotes longipronotum* n. sp.** (Coleoptera: Elateridae: Elaterinae: Agriotini), is described from Siirt province, Turkey. Photographs of the imago and the aedeagus, and drawings of the aedeagus of the new species, ***A. sameki***, ***A. bulgaricus***, and ***A. rahmei*** are given. A rearranged diagnostic key of all Turkish species of ***nuceus***-group is given. The new species is discussed in relation with closely related species. The species of the ***Agriotes nuceus***-group from Turkey are listed, and their distributions are given.

## Introduction

The genus ***Agriotes*** Eschscholtz (Coleoptera: Elateridae: Elaterinae) is one of the richest genus of the tribe Agriotini Champion. According to present literature (Mertlik and Platia 2008; Platia 2008, 2010, 2011, 2012; Kabalak and Sert 2009, 2011; Platia et al. 2009, 2011; Platia and Nemeth 2011), there are 82 species of this genus in Turkey. The new species belongs to the ***nuceus*** -group of the genus ***Agriotes*** . The ***nuceus*** -group, which is separated from other species of the genus ***Agriotes*** by having the supraantennal carina reaching to the anterior margin of the frons, has 42 species distributed in Greece, Iraq, Lebanon, Syria, and Turkey (Gurjeva 1972; Platia and Gudenzi 1997; Platia 2003, 2010, 2011, 2012; Cate 2007; Platia et al. 2009, 2011; Platia and Nemeth 2011). Twenty-nine species of the ***nuceus*** -group are present in Turkey (Table 1) (Cate 2007; Platia et al. 2009; Platia 2010, 2011; Platia and Nemeth 2011).

## Materials and Methods

Specimens of the new species were collected from a pistachio (***Pistacio vera*** L.) field in Siirt province, Turkey, by using light traps. Morphological structures of the new species are described; photographs of the entire body of the male specimen, antennae, and aedeagus were taken using a Leica MZ 16A stereoscopic microscope system (www.leicamicrosystems.com) and Leica DFC320 camera attachment. The male genital organ of ***A. longipronotum* n.sp.** was pulled out.

**Table 1.**
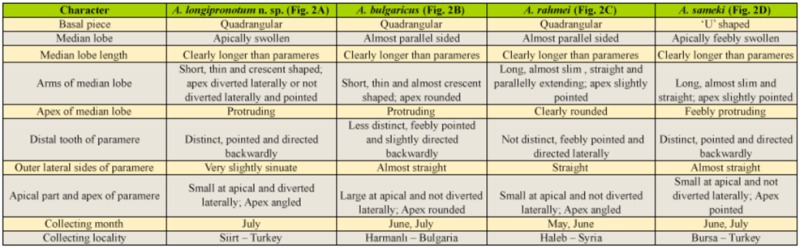
Comparison of taxonomical characters, and list of collecting month and collecting locality of some species of the ***Agriotes nuceus*** -group.

Body lengths of specimens were measured along the midline from the anterior margin of the frons to the apex of the elytra, and widths of specimens were measured across the broadest part of the elytra.

**Figure 1.**
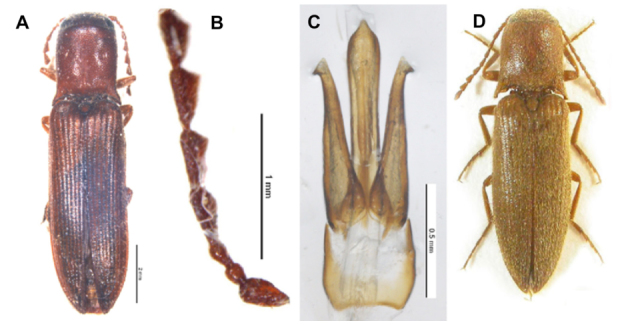
***Agriotes longipronotum* n. sp.** A. habitus male, B. antennae, C. aedeagus (scale = 0.5 mm), D. ***Agriotes sameki*** (Dusanek and Mertlik). High quality figures are available online.

**Figure 2.**
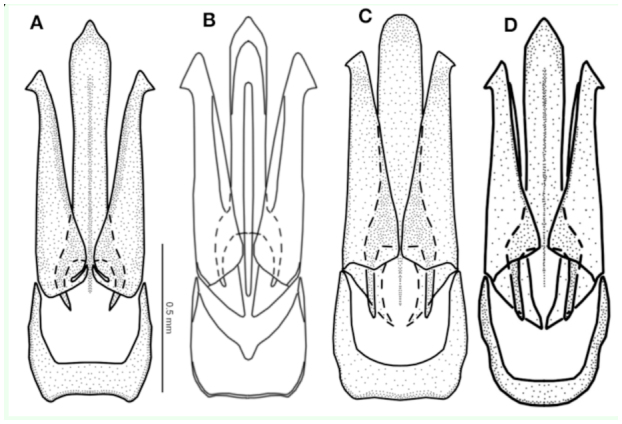
Aedeagus drawings of species. A. ***A. longipronotum* n. sp.** (scale = 0.5 mm), B. ***A. bulgaricus*** (drawn from Platia and Gudenzi 2007), C. ***A. rahmei*** (drawn from Platia and Nemeth 2011), D. ***A. sameki*** (drawn from Platia 2003). High quality figures are available online.

General morphology of the new species was compared with *A. **sameki*** Platia (Figure 1D), which is a closely related species based on its general appearance. Male genital organs of ***A. longipronotum* n. sp.** (Figure 2A), ***A. sameki*** and its closely related species (***A. bulgaricus*** and ***A. rahmei*** ) are given and are compared in Table 1. Male genital organ drawings of ***A*. *bulgaricus*** (Figure 2B), ***A. rahmei*** (Figure 2C), and ***A. sameki*** (Figure 2D) were redrawn from Platia (2003), Platia and Gudenzi (2007), and Platia and Nemeth (2011). The new species, ***A. borowieciorum*** Platia, Schimmel, and Tarnawski, ***A. constrictus*** Reitter, ***A. doboszi*** Platia, Schimmel, and Tarnawski, ***A. gulnariensis*** Platia, ***A. hatayensis*** Platia, and *A. **podlussanyi*** Platia and Nemeth were inserted into the diagnostic key of Platia (2003) in order to update the identification key to the known species of the ***Agriotes nuceus*** group of Turkey (males).

**Table 2.**
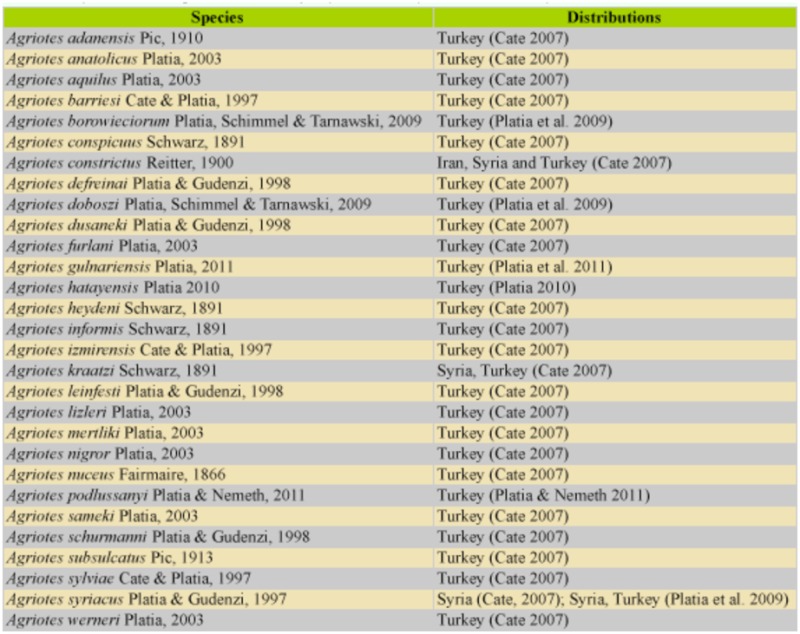
The species of the ***Agriotes nuceus***-group from Turkey and their currently known distribution.

**Figure 3.**
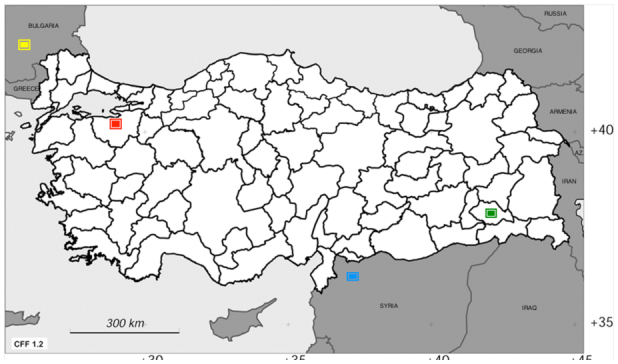
Distribution map of species made in Carto Fauna-Flora. Green mark. ***Agriotes longipronotum*** n. sp. (Siirt, Turkey), Red mark. ***A. sameki*** (Bursa, Turkey), Yellow mark. ***A. bulgaricus*** (Harmanli, Bulgaria), Blue mark. ***A. rahmei*** (Haleb, Syria). High quality figures are available online.

Distribution map of ***A. longipronotum* n. sp.**, ***A. sameki*** , ***A. bulgaricus*** , and ***A. rahmei*** was made on Carto Fauna-Flora (Barbier and Rasmont 1996, 2000; Figure 3). All species and their distributions of the ***Agriotes nuceus*** -group of Turkey are given in Table 2.

### Taxonomy

***Agriotes longipronotum* n. sp. (1A, B)**
**Type Locality:** Holotype, 1 ♂, Siirt province, 01 July 2009, leg. İ. Özgen. Paratypes, 2 ♂♂, Siirt province, 01 July 2009, leg. İ. Özgen. The holotype and one of the paratype are deposited in Hacettepe University Zoology Museum at Hacettepe University Biology Department Ankara, and the other paratype is deposited in the collection of Dr. Giuseppe Platia in Gatteo, Italy.
**Holotype:** Male. Moderately shiny; body entirely ferruginous; covered with dense, yellow pubescence.
Frons flat, slightly impressed at anterior part, anterior margin straight, suprantennal carinae not reaching anterior part, punctures umbilicate, contiguous.
Tenth and last antennal segments broken off due to the length of ninth segment, the antennae look like they exceed the apices of the posterior angles of the pronotum by about one segment, serrate from fourth segment on. Second and third segments small, second subcylindrical 1.15 times longer than wide, third subconical 1.16 times longer than second, second with a fairly larger diameter; second and third, taken together, clearly shorter than fourth, fourth to ninth triangular, longer than wide, gradually tapering.
Pronotum 1.1 times longer than wide, widest at posterior angles, strongly convex, abruptly sloping at sides, sloping more gradually at base, with a short and distinct median longitudinal depression on basal declivity; sides briefly subparallel in middle, dilated in anterior third, sinuate before posterior angles, the latter rather acute, diverging, with a moderate, apparent, inwards oriented carina; lateral suture curved, directed to lowerside of eyes, briefly obsolete near middle, punctation rather uniformly distributed, punctures on disc deep, simple to slightly umbilicate, with intervals longer than their own diameters, gradually denser towards sides, laterally contiguous to confluent.
Scutellum tongue-shaped, flat, densely punctured.
Elytra as wide as base of pronotum, elytra 2.5 times longer than pronotum, 2.8 times longer than wide, sides subparallel in the anterior 2/3 part than gradually tapering to apex, striae well marked and punctured, interstriae flat, with rough surface; prosternai sutures briefly furrowed in front.
Female unknown.
**Holotype Size:** Length 10.28 mm; width 2.57 mm.
**Etymology:** The name is derived from the length of pronotum.
**Paratype:** 2 ♂♂, length 9.62–9.70 mm; width 2.42–2.43 mm, body color of paratypes same as holotype. Apex of arms of median lobe diverted laterally in one of paratypes.
**Structure of aedeagus (dorsal view) (Figure 1C, 2A) (length 1.29 mm):** Lateral of basal part widest at medial, posterior margin arcuately concave, anterior margin U-shapedly notched, sides of basal part strongly, rest part slightly chitinized; median lobe clearly longer than parameres, feebly chitinized except medially extending strongly chitinized line, median lobe bullate apically, apex of median lobe protruded, arms of median lobe short, thin, crescent shaped, and pointed at apex; outer lateral sides of parameres feebly sinuate, distal teeth distinct, pointed and directed laterally, parameres angled at apex.
In the present study, a new species belonging to the ***nuceus*** -group of the genus ***Agriotes*** is described. ***A. longipronotum* n. sp.** is easily separated from all known species of the ***A. nuceus*** -group from Turkey by the pronotum, which is 1.1 times longer than wide. According to the morphology of the antennae and the aedeagus, the new species is closely related to ***A. sameki*** . The new species can be separated by the following combination of features: the body length of ***A. longipronotum* n. sp.** is longer than ***A. sameki*** ; the ratio of elytra/pronotum lengths of ***A. longipronotum* n. sp.** is smaller than ***A. sameki*** ; the pronotum is longer than wide in the new species while it is as long as wide in ***A. sameki*** . A comparison of the taxonomical characters, and a list of the collecting month and locality, of ***A. longipronotum* n. sp.**, ***A. sameki*** , ***A. bulgaricus*** , and ***A. rahmei*** are given in Table. 1.
***Agriotes rahmei*** can be easily separated from ***A. longipronotum* n. sp.**, ***A. sameki*** , and ***A. bulgaricus*** by having a clearly rounded apex of the median lobe and by not having a distinct distal tooth of the paramere. The aedeagus of the new species have similarities with both ***A. bulgaricus*** and ***A. sameki*** . ***Agriotes longipronotum* n. sp.** is close to ***A. sameki*** by having small parameres apically, distinct, paramere with a pointed and directed backwardly distal tooth; it is also close to ***A. bulgaricus*** in having a quadrangular basal piece and protruding apex of the median lobe. ***Agriotes longipronotum* n. sp.** can be sepa- rated from ***A. bulgaricus*** and ***A. sameki*** by the presence of a distinctly swollen apical part of the median lobe, very slightly sinuate outer lateral margin, and small and laterally diverted apical part of parameres.

**Key to the known species of *Agriotes* of the *nuceus* group from Turkey (males)**

**1**. Pronotum (included apices of posterior angles) longer than wide ***longipronotum*** n. sp.

**1**′. Pronotum (included apices of posterior angles) as long as wide **2**

**1**″. Pronotum (included apices of posterior angles) wider than long **6**

**2**. Frons not impressed before the anterior margin **3**

**2**′. Frons impressed before the anterior margin ***schurmanni*** Platia and Gudenzi 1998

**3**. Body size smaller (length 9–9.5 mm; width 2.5–2.8 mm) **4**

**3**′. Body size larger (length 11.8–16 mm; width 3–4 mm **5**

**4**. Second antennal segment longer than wide; pronotal disk convex ***sameki*** Platia 2003

**4**′. Second antennal segment as long as wide; pronotal disk depressed ***subsulcatus*** Pic 1913

**5**. Longer antennae with second and third articles globose, as long as wide ***borowieciorum*** Platia, Schimmel, and Tarnawski 2009

**5**′. Shorter antennae with second and third articles slenderer, second subcylindrical, third subconical ***furlani*** Platia 2003

**6**. Second and third antennal segments taken together shorter than fourth **7**

**6**′. Second and third antennal segments taken together as long as fourth **13**

**6**″. Second and third antennal segments taken together longer than fourth **22**

**7**. Longer antennae exceeding by more than 2.5 segments the apices of posterior angles of pronotum **8**

**7**′. Shorter antennae exceeding at best by 2 segments the apices of posterior angles of pronotum **9**

**8**. Color yellowish; body smaller (length 11.2 mm; width 3 mm); longer antennae exceeding by 4 segments the apices of posterior angles of pronotum ***izmirensis*** Cate and Platia 1997

**8**′. Color ferruginous; body larger (length 13– 15 mm; width 3.5–4 mm); shorter antennae exceeding by 2.5–3 segments the apices of posterior angles of pronotum ***heydeni*** Schwarz 1891

**9**. Body on average narrower (width 3–3.7 mm) **12**

**9**′. Body on average wider (width 3.9–4.2 mm) **10**

**10**. Second antennal segment subcylindrical, third antennal segment subconical **11**

**10**′. Second and third antennal segments subcylindrical; color blackish ***anatolicus*** Platia 2003

**11**. Elytra 3 times longer than pronotum; body color ferruginous ***podlussanyi*** Platia and Nemeth 2011

**11**′. Elytra 2.9 times longer than pronotum; body color dark brown ***gulnariensis*** Platia 2011

**12**. Pronotal sides concave in the median part ***mertliki*** Platia 2003

**12**′. Pronotal sides subparallel in the median part ***werneri*** Platia 2003

**13**. Third antennal segment subconical, longer than wide **14**

**13**′. Third antennal segment subtriangular, as long as wide ***leinfesti*** Platia and Gudenzi 1998

**14**. Body size larger (length 12.5–15.5 mm; width 3.37–4.5 mm) **15**

**14**′. Body size smaller (length 9–10.7 mm; width 2.6–3.1 mm) **19**

**15**. Longer antennae exceeding by 2.5 segments the apices of posterior angles of pronotum ***hatayensis*** Platia 2010

**15**′. Shorter antennae exceeding by two segments the apices of posterior angles of pronotum **16**

**15**″. Shorter antennae exceeding by 1–1.5 segments the apices of posterior angles of pronotum **17**

**16**. Lateral margins of pronotum complete ***conspicuus*** Schwarz 1891

**16**′. Lateral margins of pronotum interrupted at middle ***kraatzi*** Schwarz 1891

**17**. Body narrower (width 3.37–4.0 mm); color variable **18**

**17**′. Body wider (width 4.4–4.6 mm); color blackish ***nigror*** Platia 2003

**18**. Body color darker; second antennal segment less slender, normally as long as wide or just longer than wide ***doboszi*** Platia, Schimmel, and Tarnawski 2009

**18**′. Body color lighter; second antennal segment cylindirical, slightly longer than wide ***lizleri*** Platia 2003

**19**. Color yellow ferruginous **20**

**19**′. Color brown ferruginous ***dusaneki*** Platia and Gudenzi 1998

**20**. Longer antennae exceeding by about 3 segments the apices of posterior angles of pronotum - ***adanensis*** Pic 1910

**20**′. Shorter antennae exceeding by 1.5–2 segments the apices of posterior angles of pronotum **21**

**21**. Pronotum with short basal midlongitudinal furrow; scutellum tongue-shaped; elytra 2.8–3.0 times longer than pronotum and elytra 2.3 times longer than wide ***barriesi*** Cate and Platia 1997

**21**′. Pronotum without short basal midlongitudinal furrow; scutellum mitriform; elytra 3.3 times longer than pronotum and elytra 2.7 times longer than wide ***constrictus*** Reitter 1900

**22**. Second antennal segment a little longer than third **23**

**22**′. Second antennal segment a little shorter than third ***nuceus*** Fairmaire 1866

**22**″. Second and third antennal segments subequal ***aquilus*** Platia 2003

**23**. Larger species (length 14–16 mm; 4–4.8 mm) ***informis*** Schwarz 1891

**23**′. Smaller species (length 10.8–11 mm; 4– 4.8 mm) ***defreinai*** Platia and Gudenzi 1998

## Discussion

Collecting months, collecting localities, and distributions of the species of ***Agriotes*** ***nuceus***-group are listed according to the literature (Platia 2003; Platia and Gudenzi 2007; Platia and Nemeth 2011) (Table 1). Species are present in nature from May to July. Only ***A. longipronotum* n. sp.** has been collected in one month (July). ***Agriotes sameki*** (in Bursa) and ***A. longipronotum* n. sp.** (in Siirt) are present in Turkey. ***A. bulgaricus*** (Bulgaria-Harmanli) and ***A. rahmei*** (Syria-Haleb) are not recorded from Turkey.
